# High‐throughput behavioral phenotyping of tiny arthropods: Chemosensory traits in a mesostigmatic hematophagous mite

**DOI:** 10.1002/jez.2651

**Published:** 2022-09-02

**Authors:** Stefano Masier, Adrien Taudière, Laurent J. M. Roy, David Carrasco, Jean‐Yves Barnagaud, Camille Planchon, Anne‐Sophie Soulié, Nathalie Sleeckx, Lise Roy

**Affiliations:** ^1^ CEFE, Univ Montpellier, CNRS, EPHE, IRD, Univ Paul Valéry Montpellier 3 Montpellier France; ^2^ BTS CIRA Saint‐Genis‐Laval France; ^3^ MiVEGEC, University of Montpellier, IRD, CNRS Montpellier France; ^4^ Experimental Poultry Centre Geel Belgium

**Keywords:** automatic tracking, chemical ecology, ethomics, hematophagous mites, poultry red mites

## Abstract

Pest management using attractive and/or repellent semiochemicals is a key alternative to synthetic insecticides. Its implementation requires a good understanding of the intra‐ and interspecific chemical interactions of arthropod pests, their interactions with their abiotic environment, as well as their evolutionary dynamics. Although mites include many pest species and biocontrol agents of economic importance in agriculture, their chemical ecology is largely understudied compared to insects. We developed a high‐throughput ethomics system to analyze these small arthropods and conducted a study on *Dermanyssus gallinae*, a problematic poultry parasite in the egg industry. Our purpose was to elucidate the role played by host‐derived odorants (synthetic kairomone) and conspecific odorants (mite body odors) in *D. gallinae*. After validating our nanocomputer controlled olfactometric system with volatile semiochemicals of known biological activity, we characterized response traits to kairomonal and/or pheromonal volatile blends in mites from different populations. We were able to accurately characterize the repulsion or attraction behaviors in >1000 individual specimens in a standardized way. Our results confirm the presence of a volatile aggregation pheromone emitted by *D. gallinae* and bring new elements to the effect of odor source presentation. Our results also confirm the attractive effect on *Dermanyssus gallinae* of a blend of volatile compounds contained in hen odor, while highlighting a repellent effect at high concentration. Significant interindividual and interpopulation variation was noted particularly in responses to synthetic kairomone. This information lays a valuable foundation for further exploring the emergence risk of resistance to semiochemicals.

## RESEARCH HIGHLIGHTS

Through a new high‐throughput system based on nanocomputers connected to cameras automatically tracking movements, we highlight behavioral responses of a hematophagous mite species to kairomones and pheromones.

## Graphical abstract

We validated a new high‐throughput system based on nanocomputers connected to cameras automatically tracking movements of single mites. Our results confirm the existence of a volatile pheromone deposited by *D. gallinae* and attractive to conspecifics. They also show that a mixture of volatile compounds contained in the hen odor is attractive at low concentrations and repulsive at higher concentrations.

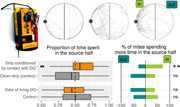

## 1 INTRODUCTION

Studies of the chemical ecology of target pests, including the evolving capacity of chemosensory traits, address two of the central principles of integrated pest management (IPM) (Barzman et al., 2015). First, such studies may significantly help to reduce the use of pesticides by opening up avenues for the development of alternative or complementary semiochemical‐based control techniques (Blassioli‐Moraes et al., 2019; Mbaluto et al., 2020; Weeks et al., 2013). Second, as with any pest control measure, the semiochemicals used in IPM strategies may exert a selective pressure that can lead to the emergence of resistance. A few pioneering studies show that resistances to repellents have indeed developed in insect populations (Deletre et al., 2019; Mengoni & Alzogaray, 2018; Stanczyk et al., 2010; Vassena et al., 2019; Yang et al., 2020). To our knowledge, resistance to attractants has not yet been studied. Assessing the variation of responsive and unresponsive phenotype frequencies among populations and their heritability based on standardized bioassays, as is done for pesticide resistance, would satisfy anti‐ resistance strategies, another central principle of IPM (Barres et al., 2016; Barzman et al., 2015). It is thus crucial to unravel chemical interactions and the evolution of their chemosensory traits in arthropods to successfully implement IPM strategies. However, such studies have so far focused on insects, although Acari (mites and ticks) species of agricultural, veterinary or medical interest are numerous, both as crop and livestock pests (e.g., Attia et al., 2013; Lihou et al., 2020; Shahid et al., 2021; Sparagano et al., 2014). As most of them have no sense of sight, their behavior is likely to be strongly influenced by semiochemicals.

Interfering in intra‐ or interspecific interactions through volatile semiochemicals is among the key methods to manage pests without pesticides (Blassioli‐Moraes et al., 2019). Chemosensory detection of volatile compounds is an incredibly important mechanism for inter‐ and intraspecific communication, especially in arthropods (Gadenne et al., 2016; Koehl, 2001; Pelosi et al., 2014; Poldy, 2020), and even more in those species that cannot rely on visual cues for their decision‐making processes (Salmon et al., 2019; Zizzari et al., 2017). Individuals use volatile compounds to remotely locate conspecifics, nutrient sources, predators, or even injured conspecifics (Dicke et al., 1993; Dötterl & Vereecken, 2010; Hermann & Thaler, 2014; Johansson & Jones, 2007; Margolies et al., 1997; Richard & Hunt, 2013; Venuleo et al., 2017). Among semiochemical compounds, pheromones and allelochemicals are substances that mediate intraspecific and interspecific communication, respectively. For example, sex pheromones drive the encounters of individuals of different sexes, while aggregation pheromones allow conspecifics to group together (Hanks & Millar, 2016; Wertheim et al., 2005). Among allelochemicals, kairomones produce an advantageous reaction to the receiving species but are negative to the emitter, for example, volatile compounds produced by preys and used by predators to locate them (Dicke & Sabelis, 1988). Pest management techniques based on semiochemicals are heterogenous, for instance: plain plant‐based repellents applications help keeping hematophagous arthropods away from humans or livestock (El Adouzi et al., 2020; Pohlit et al., 2011; Rehman et al., 2014); “attract and kill” strategies rely on traps baited with pheromones or kairomones to either monitor or reduce pest infestations (Anshelevich et al., 1993; Duelli et al., 1997; McNeil, 1991; Morrison et al., 2016); “push‐pull” strategies consist of making the target crop or livestock unfriendly or inaccessible to the pest while offering a more attractive alternative (Bhattacharyya, 2017; Khan et al., 2016).

To decipher the role of semiochemicals in pest management, it is crucial to be able to link the exposure to volatile compounds to a more or less specific behavioral response through standardized tests on a large number of individuals. For instance, moving away from the odor source for a true repellent or losing the ability to orient toward an attractive source for an odor mask (Deletre et al., 2016), orienting toward the source for a tactical attractant, or increasing activity for a kinetic attractant (Miller et al., 2009). Choice tests on individual mites in controlled airflow olfactometers are very useful, but are too much time‐consuming to properly assess intra‐ and interpopulation variation (El Adouzi et al., 2020; Koenraadt & Dicke, 2010; Zeringóta et al., 2020). To speed up the process, bioassays can also be conducted on groups of individuals by sorting responsive and unresponsive individuals according to their position relative to an odor source in delimited areas (e.g., Dietrich et al., 2006; Maeda, 2005; Nachappa et al., 2010). However, intraspecific interactions and contact with the substance can then lead to confounding effects. As a workaround, we developed still‐air olfactometers driven by nano‐computers with cameras to perform standardized high‐throughput ethomic bioassays on single individuals without tactile access to the compounds source. This solution is intermediate between the commercial olfactometric tool used by Weeks et al. (2013) and the open source and cheap ethomics tool developed by Geissmann et al. (2017). Our tool is as cheap as Geissmann et al.'s system and has been specifically designed to register the movement of a nonflying, small (<2 mm) arthropod in the presence of a specific odor source in an easily‐cleanable arena allowing large movements in two dimensions.

Poultry farms in Europe and elsewhere host, with a very high prevalence, the poultry red mite *Dermanyssus gallinae* (De Geer, 1778), a hematophagous pest. *D. gallinae* causes severe disturbances and health issues in poultry, which eventually translate into high economic losses for farmers (Sparagano et al., 2014). The development of IPM against *D. gallinae* pests has been recommended for a long time, but it is still in its infancy (Decru et al., 2020). It considers, among others, making hen less attractive through feed additives based on repellent plant extracts (El Adouzi et al., 2020) and developing attract & kill strategies thanks to a patented attractive odorant blend mimicking the hens’ odor (Roy et al., 2018). Since *D. gallinae* lacks eyes, like most Mesostigmatic mites, it relies on nonvisual cues to locate its food sources (Krantz & Walter, 2009; Pritchard et al., 2015). It has been proved that this species uses, among other things, olfactory cues to orient itself (El Adouzi et al., 2020; Entrekin & Oliver, 1982; Koenraadt & Dicke, 2010).

The main objective of this study was to elucidate the role played by host‐ and mite‐emitted odorants in *D. gallinae* (DG). To do this, we first developed and validated the use of nano‐computer driven olfactometric bioassays for the study of orientation behavior and activity of mites in response to volatile compounds with opposite activities: an attractant and a repellent. Using this tool, we then characterized the biological activity of volatile compound blends with pheromonal and/or kairomonal function in *D. gallinae*.

## 2 MATERIALS AND METHODS

### 2.1 Mites under test

Tested individuals of *D. gallinae* (adult females; length: 0.75–1.5 mm) were collected from several laying hen farms: two French farms (DIN, Pierrelatte; DG‐FR10) and two British farms (DG‐UK04; DG‐UK05) to compare populations assumed to be reproductively isolated for a long time (UK vs. France) or not (within each country) (Roy et al., 2021). In this context, we consider as a population a set of individuals that live in a given farm. To obtain a representative overview of the frequency of chemosensory responses in the populations, we sampled hundreds of mites from each of the 6–10 randomly selected points within the henhouse and placed them in separate ziplock bags. For the bioassays (see below), we randomly took a balanced number of individuals from each bag.

Alive mites were then kept without food inside ziplock bags at 17°C for >48 h before testing to stimulate their activity. No individuals were kept for more than 20 days before the test, as their activity seems to drop significantly after 3 weeks of starvation (S. M. and L. R. personal observations; see also Kilpinen & Mullens, 2004).

### 2.2 Electronic olfactometer: The MiteMap system

**Figure 1:**
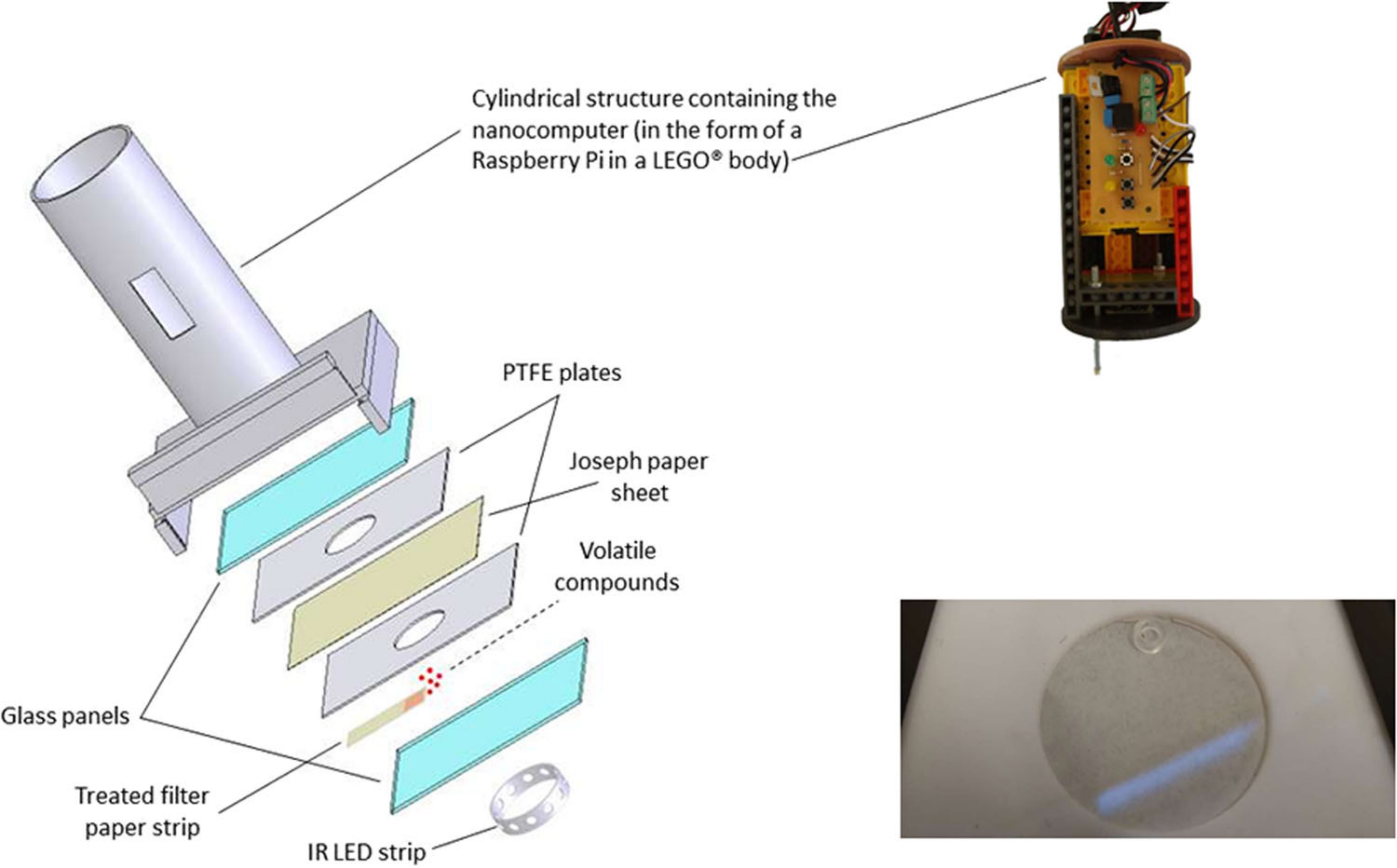
Schematic representation of the MiteMap system. The exploded view on the left represents the sandwich that forms the arena once assembled (from the top glass panel to the bottom glass panel) positioned in the lower part of the PVC chamber (tube and rectangular base, LED strip). The mite under test is enclosed in the circular hole of the upper polytetrafluoroethylene (PTFE) plate, on the Joseph paper. The main LEGO® body (top right) contains a nano‐computer and a downward facing camera. It is inserted into the chamber tube during testing, so that the camera is positioned just above it, 40 mm apart. The odor source is inserted under the underside of the Joseph paper, which acts as a floor. In many cases, this is a strip of odor‐impregnated filter paper, but it is replaced by a mite enclosed in a PTFE ring when testing the effect of odor emitted by the body of whole live mites. A photograph of the underside of an arena (bottom right) shows a small transparent PTFE ring holding a living mite whose odor diffused through the Joseph paper into the arena on the other side.

Detailed technical, mechanical and computer information is available from the MiteMap github on a Zenodo repository (Data Availability Statement). The core of the tool was an ARM processor single board nanocomputer (Raspberry Pi 3 model B, Raspberry Pi Foundation), connected to a camera (Raspberry Pi PiNoir Camera Module V2, Raspberry Pi Foundation). The system was assembled inside a body made out of LEGO® pieces and inserted into a cylindrical polyvinyl chloride (PVC) structure of approximately 20 cm of height and 4.0 cm base radius (Figure 1). The cylindrical structure was assembled on a PVC box containing the test arena (rigid plastic T‐shaped chamber, Figure 1). A threaded metal rod attached to the LEGO® body was used to keep the camera at the correct focal distance (4.0 cm) from the test arena. During analysis, the end of the rod was lying directly on the glass ceiling of the arena and was capped with a nylon nut to protect the glass. A 76.5 mm diameter ring of 940 nm infrared light‐emitting diodes (LEDs) was placed at the bottom of the PVC box below the experimental arena (40 mm diameter) to transilluminate the test arena (LED strip Solarex). The Raspberry Pi nanocomputer was programmed in Python 3 using openCV library (https://opencv.org/) to automatically: (a) detect the test arena walls, allowing therefore the standardization of the coordinate system between bioassays; and (b) locate and track the movements of the mite placed inside. The detection of the mite position in the arena was done in several steps:

– Step 1: For each frame of the video acquisition, the OpenCV function createBackgroundSubtractorMOG2() was used to remove the background. This function was configured with a history length of 100 images (history = 100), which means that the last 100 images are used to build the background model and a sensitivity of 25 (varThreshold = 25), which allows to detect small variations of luminosity when the mite is moving.
– Step 2: The opencv function findContours() returns circles containing the moving objects detected in the previous step.
– Step 3: Detected contours whose area was greater than the maximum area of a mite, or less than its minimum area were eliminated. If a single contour was detected, then mite *x*–*y* coordinates were memorized. Otherwise, the acquisition was ignored.

**Figure 2:**
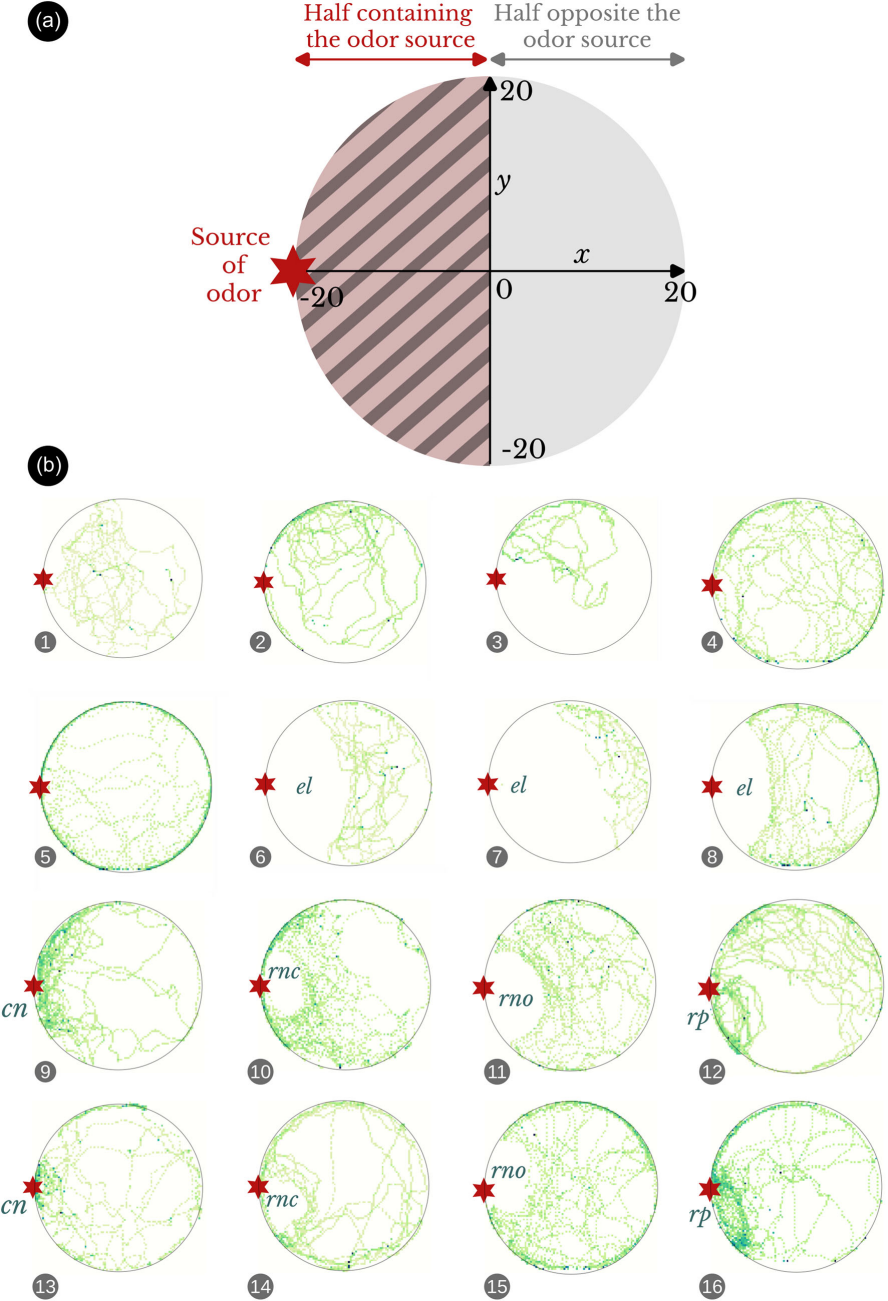
Organization of an arena and heatmaps of the path traveled inside by the tested mites. (a) Diagram of an arena with zoning by horizontal division. Half containing the source (red): all records with *x* < 0, opposite half (yellow): all records with *x* > 0. (b) Examples of individual heatmaps with or without any of the characteristic patterns defined in the present study: cn, concentrated reticulation on the source; el, eviction lens; rn, rounded notch (rno, open; rnc, closed); rp, round path on the source. The darkening of the color of the dots in the heatmaps is proportional to the time spent by the mite (the darker it is, the more time it has spent on the dot). Red star, position of the odor source.

The system automatically records the position of the mite individual every 0.2 s, creating a file with timestamp and *x*–*y* coordinates according to the diagram shown in Figure 2, as well as a Boolean variable indicating if the individual has remained immobile since the last record. From these data, the program calculates at the end of the test the following variables, based on the division by the vertical diameter: (i) Total time spent in the half containing the odor source (*x* < 0), (ii) total time spent in the opposite half (*x* > 0), (iii) time spent immobile in the half containing the odor source (*x* < 0), (iv) time spent immobile in the opposite half (*x* > 0), (v) total distance traveled in the half containing the odor source (*x* < 0) and, (vi) total distance traveled in the opposite half (*x* > 0). No pictures are stored by default to save memory space, although it is possible to collect snapshots manually or instruct the system to save a picture in specific conditions. In addition, a live feed allows us to monitor the evolution of the path followed by the mite. Two sets of images show the arena live and allow us to observe the mite itself (raw images) as well as its last 20 positions (processed images), respectively. The last 20 positions of the tested mite are visualized by drawing a tail of decreasing thickness according to the time elapsed since the current position. Lastly, a tab of the web interface allows us to visualize the heatmap of the mite's positions from the beginning of the test. The heatmap as well as all the data in csv format are downloadable.

The test arena was built through the creation of a so‐called “sandwich”: a sheet of 100% cellulose nitrate Joseph paper (23 g/m^2^; 0.07 mm thick; Ahlstrom‐Munksjö Germany GmbH) was placed between two 1.5‐mm‐thick rectangular polytetrafluoroethylene (PTFE) plates (100 × 150 mm) (Alt‐Technischer Handel GmbH) to form the floor of the experimental arena. Each plate had a circular hole in the center (40mm diameter) constituting the edges of the arena itself (Figure 1). The two PTFE plates were then inserted between two rectangular glass panels of the same dimensions, and the whole sandwich was sealed shut using two halves of 7.0 mm slide binders (ACCO Brands Europe Oxford House). This way, each side of the sandwich presents a circular‐shaped arena (40 mm diameter) with a filter paper floor, PTFE walls and a glass ceiling to allow observation and filming. On the upper side of the sandwich, the tested individual was introduced just before sealing. The lower side was used to deliver the tested substance by inserting a 10 mm large strip of Whatman filter paper between the PTFE and the lower glass panel just before introducing the mite. This strip was previously treated by impregnating one of the ends with the substance (see Section 2.3). The strip was inserted so that the treated end was exactly at the edge of the arena (red star in Figure 2). This way, no direct contact was possible between the tested individuals and the substance (i.e., the filter paper strip and the paper floor were separated from each other by the thickness of the bottom PTFE plate), while the volatile compounds were able to diffuse through the filter paper floor into the arena.

During the test, the LEGO® body was kept in position and connected to the test arena into the above described rigid plastic T‐shaped chamber. The bottom opening of the chamber was sealed with a rubber‐lined shutter to avoid any uncontrolled light stimulus. The test arena was illuminated only by the ring of infrared LED lights placed directly underneath the “sandwich.” The camera detected movement through the shadow projected by the tested individual, and the system automatically began tracking and recording when the first movement was detected inside the arena.

Nine MiteMap systems were operated at the same time (i.e., nine mite individuals were trialed at the same time, one for each device). Each nano‐computer integrated a web server that was used: (a) to monitor the experiment in each arena; (b) to retrieve data at the end of the bioassay period, in a form of. csv files (list of coordinates recorded as a function of time and variables calculated from these data according to the halves of the arena) and a. png file (heatmap of all positions); and (c) to visualize the heatmap automatically generated by the system detailing the positions of the tested individual during the trial (see MiteMap github on the Zenodo repository in Data Availability Statement). All nano‐computers in operation during the experiment were connected to a wifi access point that allowed interconnection of all nano‐computers with experimenters’ personal computer or phone.

### 2.3 Test protocol

To characterize the behavioral response of each species to each substance under test (details below and in Table 1), we performed bioassays on at least 50 individual mites separately (adult females of *D. gallinae*), including 25 test individuals (exposed to the test substance via the impregnated filter paper strip) and 25 control mites (exposed to the solvent, if any, or to an unimpregnated filter paper strip), except in two modalities. All bioassays were performed on single individuals to avoid confounding effects from intraspecific interactions, and each individual was tested once. Between the two bioassays, all the elements of the sandwich were carefully washed in 96% ethanol and air dried, with the exception of the paper floor and the filter paper strip that were both discarded.

**Table 1:** Summary of the tested substances and modalities of bioassays

Nature of substance	Synthetic			Naturally emitted by *D. gallinae*		
Bioassay	Geraniol	Ammonia (NH_3_)	Patented blend	Alive conspecifics	Liquid extract from ground conspecifics	Full nest odor
Odor source	Impregnated strip	Impregnated strip	Impregnated strip tip	1 live adult female in a PTFE ring	Impregnated strip	Strip conditioned by contact with mites
Concentration	10%	0.15% or 1.5%	0.2%, 1%, 5%, 20%, 50%, 70%, or 100%	NA	(1 or 5 mites ground in 300 µl)	NA
Solvent	Ethanol 96%	Distilled water	Dichloromethane	NA	Ethanol 96%	NA
Expected activity	Repellent (a plant secondary metabolite)	Attractant (habitat cue?)	Attractant (kairomone)	Attractant (pheromone)	Attractant (pheromone)	Attractant (pheromone)

The odor source was placed below the paper floor of the arena, right up against the circular wall of the arena to establish a strong odor gradient. It was prepared differently depending on the nature of the substance to be tested. To test synthetic compounds, a fixed solution volume of 10 µl was deposited on the strip's end. The strip was kept under ventilation in a fume hood for 30 min and was then used immediately in a bioassay (details in Table 1). We chose to insert only dry strips to avoid potential humidity gradients that could have influenced the behavior of the tested individuals, as well as to allow the solvents to evaporate and leave only the tested substances. The synthetic substances under test were geraniol (CAS no. 106‐24‐1) (98.5%; Sigma‐Aldrich), ammonia (NH_3_; CAS no. 7664‐41‐7) (NH_3_ solution 30% in distilled water; Carlo Erba) and the patented blend MIX1.0 by Roy et al. (2018), namely an equivolumic blend of (E)‐non‐2‐enal (CAS no. 18829‐56‐6), nonanoic acid (CAS no. 112‐05‐0), octanal (CAS no. 124‐13‐0), nonanal (CAS no. 124‐19‐6), oct‐1‐en‐3‐ol (CAS no. 3391‐86‐4) (respectively, 95%, 95%, 99%, 97%, 98%; Sigma‐Aldrich). Solvents used were either 96% GPR RECTAPUR® ethanol (VWR International SAS), distilled water (Proamp® water for injections; Laboratoire AGUETTANT) or dichloromethane (≥99.8% stabilized, PESTINORM® for pesticide residue analysis, VWR) (see Table 1). For substances naturally emitted by *D. gallinae*, we exposed the test mites to the odor emitted by a filter paper that had been previously in contact with mites (“full nest odor”), or to the odors emitted by the body of either whole or crushed mites. To condition filter paper strips with “full nest” odor, we placed them for 4 days in a ziplock bag containing aggregates of *D. gallinae* collected at least 7 days before the experiment. To emit the whole body odor of a living adult female in the arena, we replaced the filter paper strip with a PTFE ring (external diameter: 6 mm; internal diameter: 4 mm; 4 mm height) placed between the outer face of the paper floor and the bottom glass plate, against the edge of the circle of the bottom PTFE plate (Figure 1). Finally, to approach one of the methods used by Enterkin and Oliver (1982) to reveal the aggregation pheromone, we ground one to five mites in 96% ethanol.

The bioassay was started as follows: the odor source (i.e., the impregnated strip or the mite placed in a PTFE ring) was inserted between the lower glass plate and the lower PTFE plate of the “sandwich” as described above. A living adult female mite was then introduced into the arena (i.e., circular hole in the upper PTFE plate) using a thin paintbrush by gently shifting the upper glass plate, then the whole sandwich was quickly aligned and sealed. The sandwich was inserted into the test chamber, which was then closed as described above. The Python program was launched via the buttons on the MiteMap. Each bioassay lasted exactly 10 min starting from the first movement detected by the camera inside the arena and was checked via the web interface. We checked the correct positioning of the virtual arena boundaries on the real arena via the live feed at the beginning of each test for all MiteMaps (MiteMap interrupted in case of mismatch and restarted after repositioning). In the case of prolonged inactivity at the beginning of the bioassay (i.e., no start of the recording for >2 min), the mite was discarded and replaced by another one. The substances tested inside each MiteMap system were randomized between bioassays.

### 2.4 Tested volatile compounds

#### 2.4.1 Validation of the MiteMap olfactometric system

For the validation of the system, we have carried out preliminary experiments during which we have evaluated the risk of signal loss or other recording defects during the test using the live feed. To do this, we reviewed the entire live feed of 20 tests and identified any discrepancies. We then tested two identified volatile compounds: geraniol ((2E)‐3,7‐dimethylocta‐2,6‐dien‐1‐ol) and NH_3_ as reference odors. Their respective repellent and attractant activity on *D. gallinae* was known at the beginning of the study, based on classical olfactometer tests. Geraniol is a plant‐derived single molecule, known for its repellent effect on *D. gallinae* (Soulié et al., 2021) and commonly used along with other molecules for commercially distributed repellents. NH_3_ is known to have an attractant effect on hematophagous arthropods (Geier et al., 1999; Haggart & Davis, 1980; Sukumaran, 2016; van Loon et al., 2015) and some nonhematophagous insects (Abuin et al., 2011; Kendra et al., 2005; Min et al., 2013), mostly as a probable cue to locate food sources. We observed its attractive effect also on *D. gallinae* in previous Y‐tube olfactometer tests (Roy, unpublished data). We tested its effect at two different concentrations (0.15% and 1.5%) because NH_3_ can be found with very variable concentrations in different parts of the henhouses, being emitted in large amounts by microorganisms in manure (Lauer et al., 1976), but it is also likely that it is emitted in lower amounts by mites’ feces and by the hens’ body. Both molecules have known, opposite and documented effects on various arthropods, including *D. gallinae*, are easy to obtain and handle and have been extensively tested beforehand in traditional olfactometric systems (El Adouzi et al., 2020 for geraniol; unpublished data for NH_3_): as such, they constituted the perfect baseline to test the reliability of the MiteMap system.

#### 2.4.2 Characterization of mites’ responses to volatile semiochemicals

To provide information on a potentially useful product for IPM against *D. gallinae*, we further explored the attractant activity of MIX1.0, a patented synthetic blend of five identified volatile organic compounds (VOCs), which mimic the volatiles produced by hens (patent n. WO2018109417A1; FR3060258A1) (Roy et al., 2018). It is known that some substances can have attractive and repellent properties depending on the concentration for the same arthropod species (e.g., Naik et al., 2007). To refine our understanding of the properties of this synthetic blend, we characterized the response of *D. gallinae* to a wide range of concentrations, previously developed using traditional Y‐olfactometer systems (Roy et al., 2018) and in four populations from French and UK farms to account for any possible intraspecific variation. To do this, we first had to determine the concentrations of interest (i.e., attractive) by testing seven concentrations distributed over a wide range (0.2%–100% v/v) on a reference population (FR10). Indeed, the way in which volatile compounds were presented to the mite in our traditional Y‐olfactometer system based on odor loaded air streams in intermediate containers was very different from the new technique, where the source is presented via still air and from a paper support. Once the activity of the different concentrations was evaluated on our reference population, we focused on the attractive concentrations and tested only a few of the repulsive concentrations on the other populations. Therefore, the number of concentrations tested is not the same for all populations.

To advance the knowledge of the intraspecific chemical interactions between mesostigmatic mites, we tested the odor emitted by *D. gallinae* as cues to locate conspecifics in *D. gallinae* (pheromone). Groups of *D. gallinae* coalesce and produce aggregates of up to several thousands of individuals (Maurer & Baumgärtner, 1992). It has been shown that *D. gallinae* produces a currently undescribed volatile pheromone that attracts conspecifics and induces akinesis, thus allowing them to initiate aggregates, even without direct contact (Entrekin & Oliver, 1982).

As the exact composition of said pheromone has never been described so far, we were unable to test the specific compounds, and alternatively we used the whole odor of the mite body (i.e., an odor that likely includes the pheromone as well as other volatile compounds). As we also do not know from where or how the pheromone is emitted by the mite, we chose to test different ways of emitting the pheromone, ranging from an extract of ground individuals to live conspecifics to paper conditioned by prolonged contact with mite living aggregates (see Table 1).

### 2.5 Statistical analysis

We first examined the results of experiments with the two reference substances (geraniol and NH_3_) to establish how mite responses to odors are expressed in our system. Then we successively treated the results of experiments with the kairomonal patented blend and those obtained with mite odors.

We automatically grouped the heatmaps into folders by modality (script of the function available on the Zenodo repository; see below) and visually screened the individual heatmaps by modality to identify possible recurrent path patterns (qualitative approach). To characterize in a quantitative way the response of *D. gallinae* to the proposed odor in MiteMaps, we measured the proportion of time spent in the half of the arena containing the odor source (division by vertical diameter). To assess a preference for the proposed odor, we assigned each mite to a choice based on the ratio of time spent in the half containing the odor source as follows: >0.5, odor chosen, ≤0.5, odor not chosen. We then analyzed the resulting binary data set using two‐tailed binomial tests based on the null hypothesis that the probability of scores for the half containing the odor source or for the opposite half is equal to 50%. Lastly, to evaluate the effect of the odor on the general locomotor activity of the mite, we calculated the average speed during the experiment and the proportion of time spent immobile. We conducted Kruskal–Wallis tests to assess whether any significant difference (*p* < 0.05) was detectable among modalities in the three batches of data sets and pairwise Wilcoxon tests on significant batches to identify pairs with significant differences.

All analyses were performed in R, version 4.0.4 (Team R Core, 2021). The full data set and the annotated code are available on the Zenodo repository (Data Availability Statement).

## 3 RESULTS

### 3.1 Validation of the MiteMap olfactometric system and qualification of mite responses

During preliminary experiments, we estimated the risk of signal loss during recording or inconsistent signal by confronting via the web interface live feed and data recording during 25 tests (10 min sequences for one mite each). We did not observe any erratic movement of the mites during these experiments. Adding a mite to the upper surface of the sandwich produced the expected loss of signal acquisition when the mite passed over the arena, as it represented a second target. We did not observe any error in the recordings at that step. When we screened all the heatmaps, we observed a few cases of erroneous points, recorded outside the arena. Filters were applied to exclude them from the analysis.

Examination of individual heatmaps of the controls revealed heterogeneity in the amplitude and shape of the path traveled by the mites, with some remaining less mobile than others and traveling only a portion of the arena (Figure 2). The majority, however, covered a large part of the arena and often showed thigmotactic behavior (path concentrated along the edges). Among multiple path shapes, we identified five types of characteristic and recurrent patterns associated with the position of the odor source (Figure 2b): (i) an eviction lens, namely an empty lens‐shaped area corresponding to the intersection between the contour of the arena and a circle of variable diameter around the odor source, (ii) a concentrated reticulation at the odor source, (iii) a rounded empty notch at the odor source, distinct from the eviction lens by its rounded shape and smaller size, (iv) a similar notch closed by one or more mite passages along the edge of the arena, and (v) a circular path repeated several times by the mite precisely at the odor source (Figure 2b).

Eviction lenses were recorded in 77% and 76% of all mites tested with geraniol in the DIN and FR10 populations, respectively (Table 2). This is consistent with the 82% of individuals that chose the branch opposite to geraniol in previous T‐olfactometer choice tests (El Adouzi et al., 2020; test population = DIN). This path shape is therefore an indication of the known repulsion behavior in *D. gallinae* toward this substance. In mites confronted with NH_3_, the shape of the path did not show striking recurrent patterns. Even though the mite may frequent more the specific area of emission of the attractive odor (e.g., Figure 2b, 13), the shape of the concentrated reticulation on the odor source was very variable and the mite also actively frequented the whole area. No eviction lens, nor rounded notch nor circular path on the odor source was observed in the heatmaps with NH_3_.

**Table 2:** Information on the experiments carried out and qualitative assessment of the use of the arena by the mites in the different modalities tested

Data set	Farm	Modality	*n*	Source	Opposite	*p*	Estimate	95% CI	% el	% rn + rp
Reference odors	DIN	0% geraniol (control)	25	14	11	0.690	0.560	0.349–0.756	3	0
	DIN	10% geraniol	27	1	26	0.001	0.037	0.001–0.19	77	0
	FR10	0% geraniol (control)	24	15	9	0.307	0.625	0.406–0.812	3	0
	FR10	10% geraniol	24	3	21	0.001	0.125	0.027–0.324	76	3
	DIN	0.15% NH_3_	33	23	10	0.035	0.697	0.513–0.844	0	0
	DIN	1.5% NH_3_	30	23	7	0.005	0.767	0.577–0.901	0	0
	DIN	0% NH_3_ (control)	30	18	12	0.362	0.600	0.406–0.773	0	0
	FR10	0.15% NH_3_	27	22	5	0.002	0.815	0.619–0.937	0	0
	FR10	1.5% NH_3_	30	18	12	0.362	0.600	0.406–0.773	0	0
	FR10	0% NH_3_ (control)	31	14	17	0.720	0.452	0.273–0.64	0	0
Mite odors	FR10	PTFE ring (control_LiveDG)	33	17	16	1.000	0.515	0.335–0.692	0	0
	FR10	Live_DG in PTFE ring	62	24	38	0.098	0.387	0.266–0.519	0	0
	FR10	Clean strip (control)	28	11	17	0.345	0.393	0.215–0.594	0	0
	FR10	Impregnated strip	66	47	19	0.001	0.712	0.587–0.817	0	0
	FR10	Ethanol (control_extrDG)	29	16	13	0.711	0.552	0.357–0.736	0	0
	FR10	Extract of 5 DG	26	14	12	0.845	0.538	0.334–0.734	0	0
	FR10	Extract of 1 DG	29	19	10	0.136	0.655	0.457–0.821	0	0
Patented blend	DIN	0% MIX1.0 (control)	97	59	38	0.042	0.608	0.504–0.706	0	0
	DIN	0.25% MIX1.0	28	13	15	0.851	0.464	0.275–0.661	0	8
	DIN	1% MIX1.0	62	42	20	0.007	0.677	0.547–0.791	2	25
	DIN	5% MIX1.0	34	18	16	0.864	0.529	0.351–0.702	6	31
	FR10	0% MIX1.0	78	45	33	0.213	0.577	0.46–0.688	0	0
	FR10	0.25% MIX1.0	27	22	5	0.002	0.815	0.619–0.937	0	8
	FR10	1% MIX1.0	27	15	12	0.701	0.556	0.353–0.745	4	24
	FR10	5% MIX1.0	47	26	21	0.560	0.553	0.401–0.698	4	7
	FR10	20% MIX1.0	32	13	19	0.377	0.406	0.237–0.594	6	13
	FR10	50% MIX1.0	15	0	15	0.001	0.000	0–0.218	33	17
	FR10	70% MIX1.0	15	2	13	0.007	0.133	0.017–0.405	36	0
	FR10	100% MIX1.0	47	17	30	0.079	0.362	0.227–0.515	20	8
	UK04	0% MIX1.0	27	11	16	0.442	0.407	0.224–0.612	8	0
	UK04	0.2% MIX1.0	30	23	7	0.005	0.767	0.577–0.901	0	3
	UK04	1% MIX1.0	28	16	12	0.572	0.571	0.372–0.755	0	0
	UK04	5% MIX1.0	31	25	6	0.001	0.806	0.625–0.925	3	29
	UK05	0% MIX1.0	26	10	16	0.327	0.385	0.202–0.594	0	0
	UK05	0.2% MIX1.0	29	16	13	0.711	0.552	0.357–0.736	0	0
	UK05	1% MIX1.0	24	17	7	0.064	0.708	0.489–0.874	0	16
	UK05	5% MIX1.0	30	23	7	0.005	0.767	0.577–0.901	9	24

Abbreviations: *n*, number of mites tested; Source, number of individuals having chosen the half of arena containing the source of odor; Opposite, number of individuals having chosen the opposite half; *p*, *p* value from binomial tests; Estimate, binomial estimate (=ratio of time spent in the half of arena containing the source of odor); 95% CI, 95% confidence interval for the binomial estimate; % el, percentage of heatmaps with eviction lens, % rn+rp, percentage of heatmaps with rounded characteristic path (open or closed rounded notch, circular path).

**Figure 3:**
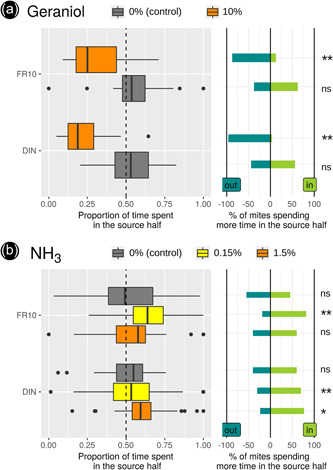
Results obtained with reference odors (populations FR10 and DIN). (a) Experiments with geraniol (solvent: ethanol). (b) Experiments with NH_3_ (solvent: water). **, binomial with *p* = 0.5 for the choice of the half of the arena containing the odor source <0.005; *, binomial <0.05; ns, binomial >0.05 (not significant).

The repellent response resulted in proportions of time spent in the half containing the odor source <0.5 in 75%–100% of the mites confronted with geraniol and the attractive response in proportions of time spent in the half containing the odor source >0.5 in ca. 75% of the mites confronted with either of the two NH_3_ concentrations tested (Figure 3). After converting the values into a Boolean choice variable, the analysis of these ratios shows a significant repellent response to geraniol, with 88% DIN mites choosing the opposite side, again consistent with previous results from classical Y‐olfactometers (Figure 3 and Table 2). Conversely, a significantly attractive response to the odor generated by the 0.15% NH_3_ concentration in both populations tested was found (Table 2). Yet, for the odor generated by the 1.5% concentration, only mites from the DIN population showed a significant preference (Table 2). We also note a slight attractive effect of ethanol (solvent for geraniol) on mites from the DIN population in the control. We did not detect any significant difference in locomotor activity (speed, time spent immobile) among modalities.

### 3.2 Characterization of mites’ responses to the patented kairomonal blend

**Figure 4:**
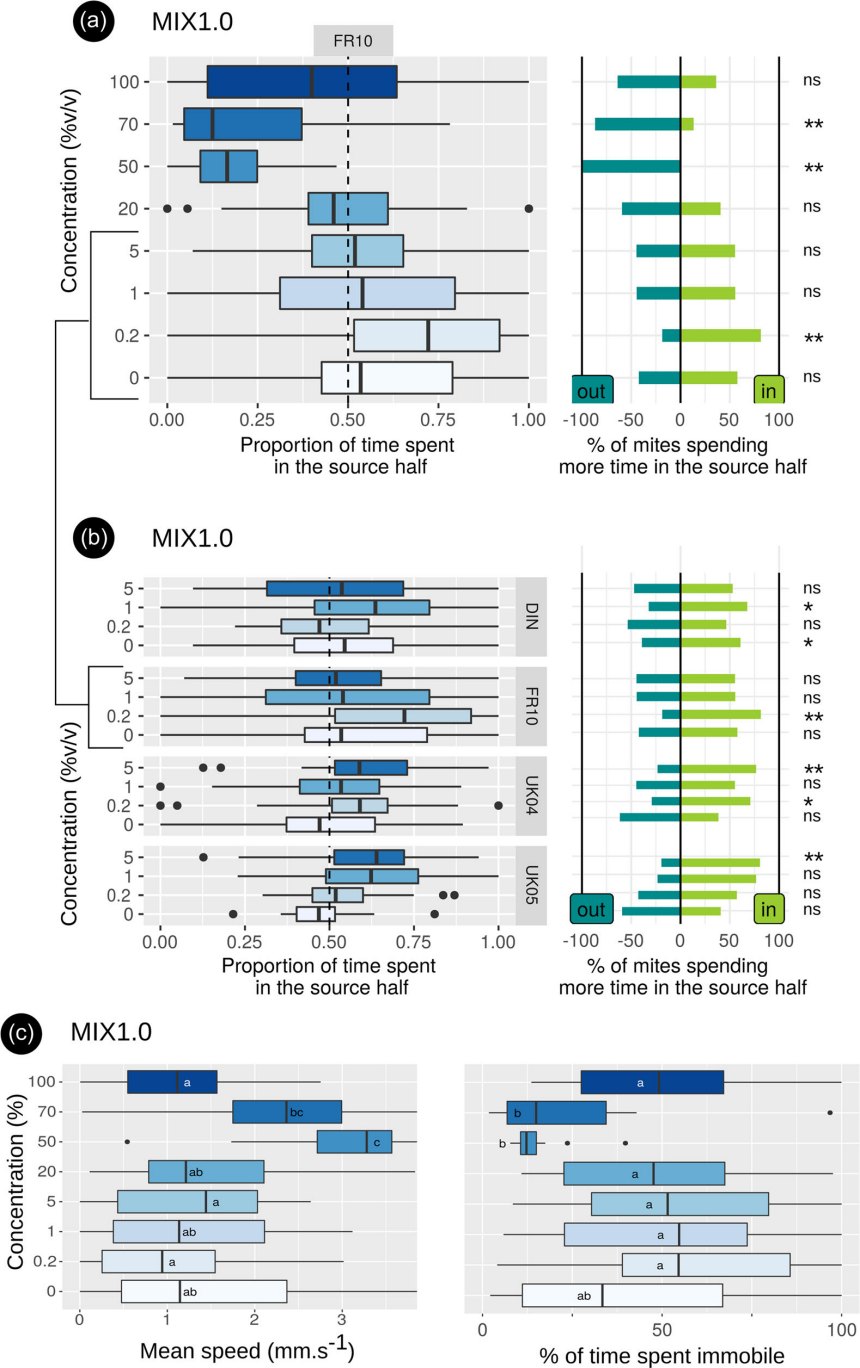
Results obtained with the patented kairomonal blend MIX1.0. (a) and (c) Experiments with population FR10 on concentrations from 0% to 100% (solvent: dichloromethane). (b) Experiments with populations FR10, DIN, UK04, and UK05 on low concentrations (the lowest concentration tested was 0.2 or 0.25 depending on the population; see Table 2). Characters indicating the results of statistical tests: rightmost characters in graphs (a) and (b): **, binomial with *p* = 0.5 for the choice of the half of the arena containing the odor source <0.005; *, ﻿﻿﻿﻿binomial <0.05; ns, binomial >0.05 (not significant); boxes of graphs (c) with different letters within a block denote significant differences according to the Kruskal–Wallis test followed by the Wilcoxon pairwise test.

The effect of the patented kairomonal blend diverged greatly depending on concentration (Figure 4): low concentrations were attractive and high concentrations repellent, with a threshold for switching from attractive to neutral or repellent varying according to the population. For the reference, French populations FR10, concentrations ≥50% showed repellent activity. The threshold from attractive to neutral/repellent was between 5% and 20% concentrations, where the distribution of individuals’ choices was not much different from 50% (Figure 4a and Table 2). This was confirmed by the recurrent presence of an eviction lens in the heatmaps (20%–36% of individuals faced with blend concentrations 50%, 70%, and 100%). Interindividual variations in the shape and magnitude of the paths were particularly marked at concentrations ≥5%: ample paths with repulsive lenses, ample paths concentrated on the side of the source and paths of reduced magnitude were recurrently recorded in single concentrations.

To assess the variation in the attractive response between populations, we studied the activity of the lowest threshold concentration (5%) and two lower concentrations in populations from two French and two UK farms. Attractive activity of the 0.2% (or 0.25%), 1%, and/or 5% concentrations was found depending on the population (Figure 4b and Table 2). In traditional olfactometers, 65% of all tested individuals (pop DIN) chose the patented blend MIX1.0 (76% of those making a choice; Roy et al., 2018). This is consistent with the 68% choice obtained here for this same population with the 1% concentration. The amplitude and significance of the measured responses were not linear: for each population, an attractive response was noted for a different concentration (FR10, 0.2%, DIN, 1%, UK, 5%), or even two concentrations separated by a concentration with no detected effect (UK04, 0.2% and 5%).

A significant increase in activity was noted for the 50% and 70% concentrations compared to the lower concentrations and to the 100% concentration (Figure 4c). In addition, even if there was no significant difference in locomotor activity between modalities, we note a markedly higherd activity of the mites of the two English populations compared to the French populations: the English populations seem to have a more regular locomotor behavior, since they were moving more slowly on average while remaining less immobile during the experiments, and this in all modalities (data not shown).

Interestingly, the rounded path patterns located at the odor source (open or closed rounded notches and circular paths) were found exclusively in the tests with patented blend MIX1.0, in 3%–31% of the individuals tested, with maximum frequencies at 1% and 5% concentrations, that is, at concentrations where the effect tends to be attractive (Table 2). We did not detect any particular association of one or the other of these patterns. They were all three recorded concomitantly, within different concentration × population modalities. Note also that eviction lenses were mixed with these patterns with most of the concentrations tested.

### 3.3 Characterization of mites’ responses to odors from their conspecifics’ body

**Figure 5:**
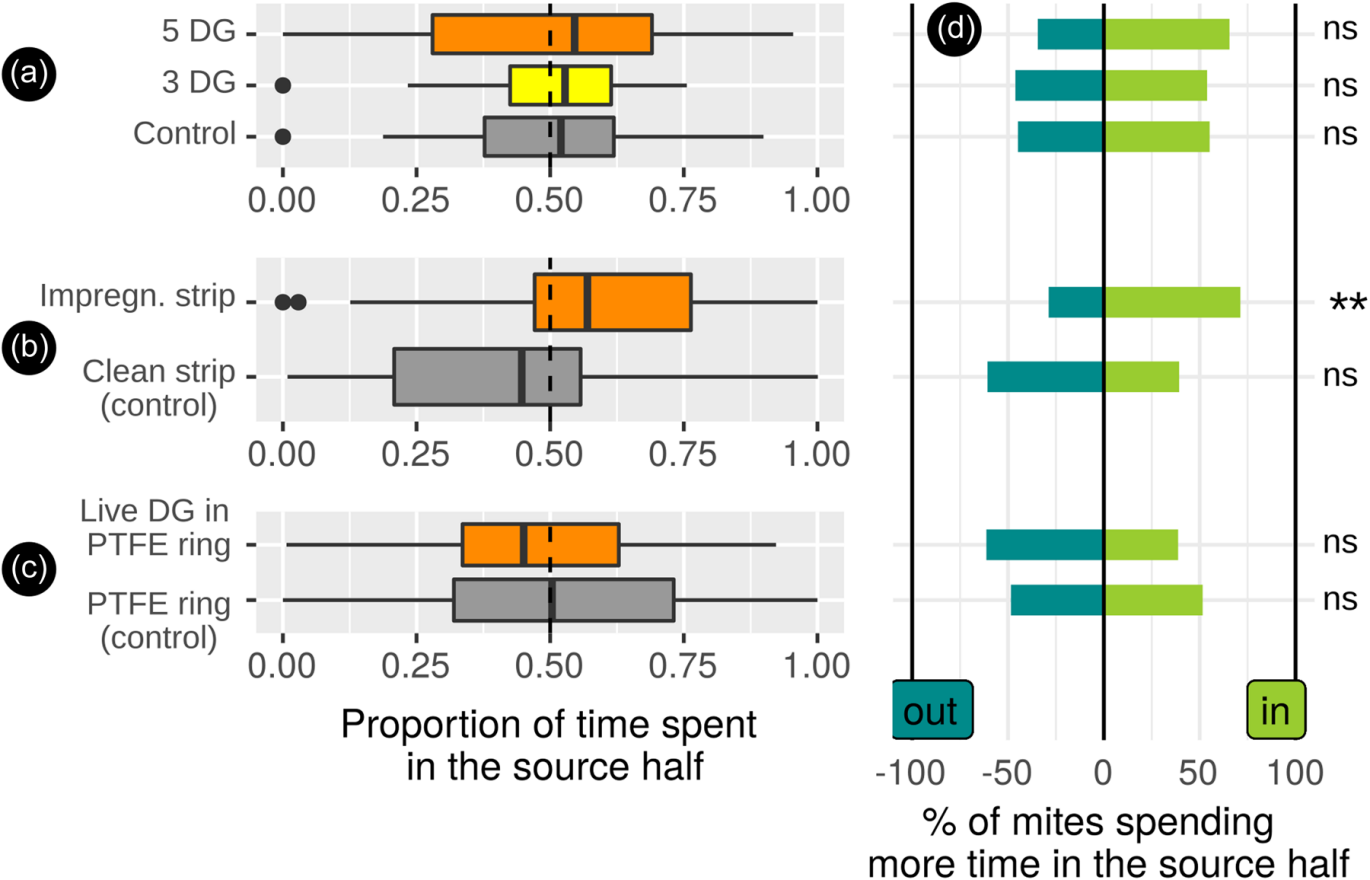
Results obtained with odors emitted by mite bodies (population FR10). (a) Experiments with mites ground in ethanol. (b) Experiments performed with single live adult females enclosed in PTFE rings. (c) Experiments performed with filter paper impregnated with the odor of mite aggregates for 4 days. **, binomial for the choice of the half of the arena containing the odor source with *p* = 0.5 < 0.005; *, binomial <0.05; ns, binomial >0.05; ns (not significant).

Regarding intraspecific communication within *D. gallinae*, we have detected different effects of the full body odor of *D. gallinae* on conspecifics depending on the emission modality. We did not detect a preference for the odor emitted by the ground *D. gallinae* extracts, nor for the odor of a living individual (Figure 5 and Table 2). In contrast, the odor emitted by filter paper impregnated with the odor of aggregates exhibited obvious attractive activity. We did not detect any significant difference in locomotor activity (speed, time spent immobile) between modalities in any experiment (data not shown). No eviction lenses or rounded path patterns were found in these tests (Table 2).

## 4 DISCUSSION

To characterize the biological activity of volatile compounds blends with pheromonal and/or kairomonal function in *D. gallinae*, we developed a nano‐computer driven olfactometric bioassay. We have verified the good agreement between the results obtained with this new tool and the traditional olfactometric systems for geraniol and the patented blend MIX1.0 on the basis of the responses measured in the mite population of the same henhouse in previous studies and the present one. The study of the responses of *D. gallinae* to different concentrations of MIX1.0 allowed us to identify a range of concentrations with repellent activity and concentrations with attractive activity slightly different according to the populations. Our results also confirmed the existence of attractive odorant deposits by mites, but do not show evidence of attractive odors passively emitted by the body of living or ground conspecifics.

### 4.1 Validation of the MiteMap system

To phenotype and study population‐level variation in the behavioral responses to different kinds of odors, it is paramount to be able to test a high number of individuals, in an efficient way and in a relatively reduced amount of time. Automatic recognition and tracking of individuals by the MiteMap system reduces the chances of human mistakes and allows the experimenter to handle multiple trials in parallel, without the need to continuously monitor every single individual as in traditional olfactometer setups. The recorded data can then be analyzed to fully characterize the behavioral response of an individual to a certain odor, as with commercial electronic tracking tools (e.g., Weeks et al., 2013), but for a lower budget. Thanks to the automation of the mite tracking, a single experimenter can operate multiple systems and perform several trials at the same time. In our study, we were able to run nine devices at the same time, performing one round of testing approximately every 30 min. In less than a month of full time work by one person, we thus recorded the chemosensory response of more than 1400 mites to several volatile compounds, the biological effects of which were known or assumed from previous studies. The design of the “sandwich” allows the animal to be confronted with odorant substances without direct contact, thus testing the response to volatile compounds only. This demonstrates the potential for high‐throughput phenotyping of chemosensory traits associated with this tool.

The consistency between the observed data and the initially expected biological activities of the two single volatile compounds tested confirms the relevance of the system. Geraniol led mites to move substantially away from the odor source and spend more time in the part of the arena a certain distance away from the odor source (eviction lens path shape, usually dozens of mm away), consistent with the expectations with true repellents (Deletre et al., 2019). Conversely, NH_3_ brought them closer without any significant effect of mite's locomotor activity, consistent with the expectations of tactical, not kinetic, attractants (Miller et al., 2009).

### 4.2 New knowledge on chemical ecology in *D. gallinae*: Effect of a kairomonal blend

Regarding the response of *D. gallinae* to the patented blend mimicking part of the hen odor, the attractiveness we had previously established in conventional Y‐olfactometers (Roy et al., 2018) was well confirmed at low concentrations based on the use of both halves of the arena. Furthermore, the rounded path patterns (round notches and circular paths), found exclusively in tests with the patented blend and especially at attractive concentrations, suggest activity centered on the odor source, just above the emission point. In hematophagous bugs with habits very similar to those of *D. gallinae*, host odors have an attractive kinetic effect early in host‐seeking activity, before acquiring an attractive tactical effect *sensu* Miller et al. (2009) (Guerenstein & Lazzari, 2009). Since the odor exposure in our test was of short duration, the local behavior observed here in part of the individuals tested could reflect a kinectic attractive effect. On the other hand, the effect of higher concentrations shows that the kairomonal blend presents a concentration threshold at which its effect switches from attractant to repellent as it has been shown with other attractant compounds (Nishimura et al., 2002; Parsons & Spence, 1981; Syed & Leal, 2009).

Interestingly, the interindividual heterogeneity of responses to different concentrations of the patented blend is large, whatever the population tested. Mites from single populations exhibit both eviction lenses and rounded characteristic paths when confronted with a given concentration. This heterogeneity probably explains the nonlinearity of responses as a function of concentration. It could come from variations in the emission of the different molecules and/or in the physiological status of the individuals. However, the preparation of the odor sources and the duration of fasting were standardized to limit this risk, and we did not observe such heterogeneity in the other tests. Given the direct link between kairomone and foraging activity, this heterogeneity could result from a mixture of genotypes determining contrasting foraging strategies within *D. gallinae* populations. For instance, the “rover” and “sitter” alleles are maintained in coexistence in natural *Drosophila* populations by frequency‐dependent negative selection (i.e., the fitness of each decreases as its frequency increases; Fitzpatrick et al., 2007). Equivalents of these alleles and their associated phenotypes have been identified in various invertebrates, with implications for the upstream functions of sensory inputs in a nematode (Fujiwara et al., 2002). It would be interesting to see to what extent interindividual variation in the olfactory response to our synthetic kairomone is associated with genetic determinants of host seeking in *D. gallinae*.

In addition, some differences among the four populations under test may be explained by the strong genetic differentiation among *D. gallinae* populations that develop on poultry farms in France and United Kingdom (Roy et al., 2021): the attractive response occurred at higher concentration in English populations than in French populations (5% vs. 0.25%–1%) and we recorded significant differences in locomotor activity between English and French populations. The extent to which concentrations of semiochemicals need to be adjusted across geographic regions may be a major issue in the implementation of IPM against *D. gallinae*. However, our study provides only a preliminary overview of interpopulation variation, as we tested a limited number of populations. A more in‐depth genetic profile of the various populations is needed to clarify the differences and state how the genetic structure may explain them. This highlights even more the importance of preliminary evaluation and monitoring to ensure that the applied concentration is appropriate (Barzman et al., 2015).

### 4.3 New knowledge on chemical ecology in *D. gallinae*: Effect of odors emitted by mites

Regarding intraspecific communication in *D. gallinae*, our results complement the data from the study by Entrekin and Oliver (1982). These authors demonstrated the existence of an aggregation pheromone that, even without contact, stimulated the creation of mite aggregates through an attractive activity coupled to akinesis induction. They obtained these results by confronting groups of living mites with the odor emitted by invisible traces deposited by groups of mites on solid supports (i.e., mites removed after impregnation, direct contact prohibited by a membrane) on the one hand, and with extracts of mites ground in a solvent on the other hand. Using filter paper impregnated with odor by live mites for several days (“full nest” odor), we detected an unambiguous attractant effect. This supports the existence of a pheromonal substance actively deposited by mites on their substrates previously shown by Enterkins and Oliver (1982).

In contrast, using an extract based on ground mite bodies similar to the extraction method of Entrekin and Oliver (1982), we did not detect any effect on the use of the arena or on the locomotor activity of the mites. There are several possible explanations for this apparent mismatch: First, our experiments tested the response by single individuals whereas Entrekin and Oliver (1982) tested the response by groups of individuals. As mites coalesce into groups after feeding for protection and reproduction, and as aggregates typically contain numerous corpses of conspecifics, it is possible that the odor cue is interpreted differently depending on the social context. Second, it is important to note that while our experiment covered only 10 min, Entrekin and Oliver (1982) maintained their setup for several hours, after which they recorded the spatial arrangement of the mites in their arena. The two apparently contradictory results may well be mere parts of a multi‐step process, where the odor emitted by the extract may evolve in such a way that it is initially overlooked and then becomes attractive. Such a process would be particularly consistent if the cue gets modified over time by natural biochemical processes (Yao et al., 2009). For instance, although the specific processes involved have not been studied, odors emitted by the corpses of conspecifics in social insects show a significant increase in fatty acid levels in the hours following death and some of these fatty acids activate burial behavior in various species (Klett et al., 2021; Wilson et al., 1958). The odor emitted by the fresh extract might either not be detected or not be associated with a situation of interest. The odor emitted after biochemical modification could be better detected and/or be a habitat/nest indicator.

Lastly, by confronting the tested mites with the odor of live mites, we did not detect any attractant effect. Given that we only use unfed mites both as odor source and test individuals, this is fully consistent with the results of Koenraadt and Dicke (2010). These authors found in Y‐olfactometers with controlled airflows that unfed mites were significantly attracted only by the odor of fed conspecifics, not by that of unfed conspecifics. All these results suggest that the mite body itself does not passively emit an attractive odor, except right after a blood meal.

With regard to pheromonal substances actively deposited by mites, the striking effect found with “full nest”‐impregnated paper strips suggests that the attractive compounds involved in the aggregation of *D. gallinae* are not directly emitted by droppings, contrary to what has been shown in hematophagous bugs with very similar habits (Gries et al., 2015; Lorenzo Figueiras et al., 1994; Mendki et al., 2014; Weeks et al., 2020). Indeed, mites emit droppings only in the hours following the blood meal (L. R. personal observations). However, the paper strips were inserted into the bags for impregnation after more than a week following the sampling on the farm and remained without spots after this process. Our study does not allow us to determine if the attractive response was due to the accumulation of volatile compounds released by byproducts of aggregated mites (fecal material, discarded egg shells and exuviae or others), to actively deposited substances or to a combination of both. Nevertheless, the convergence of our results on mite odors with those of Enterkin and Olivers (1982) and Koenraadt and Dicke (2010) argues for the action of active deposits.

It must also be noted that, in the present study, the tested individuals belonged to the same population as the mites that produced the test odor (impregnated paper strip). An interesting question arises: how do mite individuals respond to the “full nest” odor of a different population? Population‐specific, or even kin‐specific signals in the “full nest” odor may well be attractive only for those individuals that share the same genetic or social background. Alternatively, if the full nest odor is found an attractant regardless of the population of origin, it could then be possible to use the volatiles as an odorant bait for traps that could replace or complement the baits based on the above patented blend.

## 5 CONCLUSION

Besides demonstrating the suitability of the MiteMap system to characterize chemosensory phenotypes in mites, our results show how volatile cues can elicit a wide variety of behavioral responses in *D. gallinae*. This new knowledge helps to fill important gaps reported as impediments to progress in the management of *D. gallinae* in the DISCONTOOLS database (https://www.discontools.eu/database/112-poultry-red-mite.html). Furthermore, while the general profile of said responses seems conserved across populations, our results suggest potential interpopulation variations in their magnitude, or the concentration threshold needed to obtain a specific behavior. If behaviors differ significantly among farms, it will be necessary to consider the development of different control or monitoring solutions adapted to the farm or geographical area to develop IPM strategies integrating semiochemicals in a relevant way. In addition, a better understanding of the genetic determinants and consequences of the interindividual variation of responses to kairomone is a major issue to take advantage of such attractant in farm conditions: if indeed the response oscillates between attractive and repulsive within populations due to co‐occurrence of contrasting foraging strategies, setting the effective concentration will require taking into account the ratio of responses. Hence, fine‐tuning IPM treatments in each farm (Barzman et al., 2015) would be required to reach a heightened efficacy in alternative ways to control *D. gallinae* infestations in poultry farms. However, more data are still needed before being able to properly describe interpopulation variability. In addition, it will be wise to evaluate the interactions between molecules in the odor landscape of henhouses.

Furthermore, the high‐throughput phenotyping capability of our system opens up valuable perspectives for the study of the evolution of chemosensory response traits in tiny invertebrates. Since the chemosensory phenotypes of many individuals can be characterized and the phenotyped mites can be isolated at the end of the test for genotyping, this system allows not only to evaluate the intra‐ and interpopulation variation in phenotype frequency, but also their heritability. As natural selection operates on existing variation, if the observed interindividual variation is heritable, our results suggest that resistance to control or monitoring techniques based on semiochemicals may emerge more or less rapidly on farms. Therefore, to support the development of IPM against *D. gallinae*, it becomes urgent to evaluate the risk of emergence of such resistances, which are currently understudied.

## AUTHOR CONTRIBUTIONS

**Lise Roy**: Conceptualization (lead); data curation (equal); formal analysis (equal); project administration (equal); supervision (lead); validation (equal), writing – review & editing (equal). **Anne‐Sophie Soulié**: Conceptualization (supporting); investigation (equal). **David Carrasco**: Conceptualization (supporting); formal analysis (supporting); validation (equal); writing – review & editing (supporting). **Adrien Taudière**: Data curation (equal); formal analysis (equal); visualization (lead); writing – review & editing (equal). **Jean‐Yves Barnagaud**: Formal analysis (supporting); writing – review & editing (supporting). **Nathalie Sleeckx**: Funding Acquisition (lead); project administration (equal); **Camille Planchon**: Investigation (equal). **Stefano Masier**: Investigation (equal); writing – original draft preparation (lead); **Laurent J. M. Roy**: Methodology (MiteMap development) (lead); software (lead); validation (equal); writing – review & editing (supporting).

## CONFLICT OF INTEREST

The authors declare no conflict of interest.

## Data Availability

The three following resources are hosted on the Zenodo Open Science repository:
–
*MiteMap github*: Technical information, Python script and technical drawings about the electronic olfactometer 10.5281/zenodo.6916096 URL: https://zenodo.org/record/6916096#.YuGVBVTP0VA
–
*R code*: code written in RMarkdown format for all the statistical analyses carried out and the edition of the figures presented in the article, as well as code written in R format containing the functions specifically developed for our study and used in the previous code 10.5281/zenodo.6944133 URL: https://zenodo.org/record/6944133#.Yvp4FlTP1gZ
–
*Raw data*: Here are provided three groups of data sets, corresponding respectively to the data obtained (1) with the monomolecular reference substances (geraniol and NH3), (2) with the patented blend MIX1.0, (3) with the odors emitted by the mites' bodies. A folder is also provided with the heatmaps associated with these data grouped in subfolders by farm x modality 10.5281/zenodo.6944134 URL: https://zenodo.org/record/6944134#.Yvp1flTP1gZ
